# Non-genetic photoacoustic stimulation of single neurons by a tapered fiber optoacoustic emitter

**DOI:** 10.1038/s41377-021-00580-z

**Published:** 2021-07-14

**Authors:** Linli Shi, Ying Jiang, Fernando R. Fernandez, Guo Chen, Lu Lan, Heng-Ye Man, John A. White, Ji-Xin Cheng, Chen Yang

**Affiliations:** 1 Department of Chemistry, Boston University, 580 Commonwealth Avenue, Boston, MA 02215 USA; 2 Department of Biomedical Engineering, Boston University, 44 Cummington Mall, Boston, MA 02215 USA; 3 Center for Systems Neuroscience, Boston University, 610 Commonwealth Ave, Boston, MA 02215 USA; 4 Neurophotonics Center, Photonics Center, Boston University, 8 St. Mary’s Street, Boston, MA 02215 USA; 5 Department of Electrical and Computer Engineering, 8 St. Mary’s Street, Boston, MA 02215 USA; 6 Department of Biology, Boston University, 5 Cummington Mall, Boston, MA 02215 USA

**Keywords:** Biophotonics, Photoacoustics

## Abstract

Neuromodulation at high spatial resolution poses great significance in advancing fundamental knowledge in the field of neuroscience and offering novel clinical treatments. Here, we developed a tapered fiber optoacoustic emitter (TFOE) generating an ultrasound field with a high spatial precision of 39.6 µm, enabling optoacoustic activation of single neurons or subcellular structures, such as axons and dendrites. Temporally, a single acoustic pulse of sub-microsecond converted by the TFOE from a single laser pulse of 3 ns is shown as the shortest acoustic stimuli so far for successful neuron activation. The precise ultrasound generated by the TFOE enabled the integration of the optoacoustic stimulation with highly stable patch-clamp recording on single neurons. Direct measurements of the electrical response of single neurons to acoustic stimulation, which is difficult for conventional ultrasound stimulation, have been demonstrated. By coupling TFOE with ex vivo brain slice electrophysiology, we unveil cell-type-specific responses of excitatory and inhibitory neurons to acoustic stimulation. These results demonstrate that TFOE is a non-genetic single-cell and sub-cellular modulation technology, which could shed new insights into the mechanism of ultrasound neurostimulation.

## Abstract (web summary)

A tapered fiber optoacoustic emitter enables non-genetic single neuron and sub-cellular stimulation. It also offers high compatibility with stable patch clamp recording for further study of mechanism.

## Graphical abstract

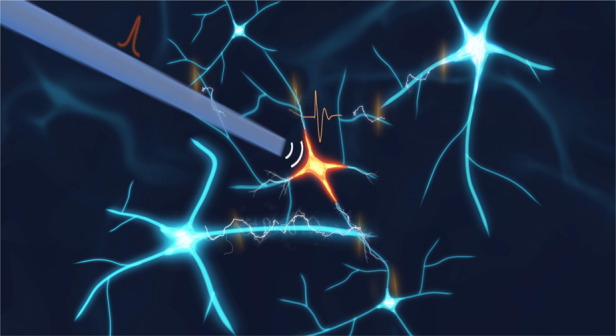

## Introduction

Neuromodulation at high spatial precision poses great significance in advancing fundamental knowledge in the field of neuroscience, as the firing of a small population or even single neurons can specifically alter animal behavior or brain state^1,2^. Clinically, precise neural stimulation lays the foundation for procedures such as retinal stimulation^3,4^ and selective dorsal rhizotomy (SDR)^5^, where selective activation of a small population or single neurons and axon fibers is desired. Historically, electrical stimulation has been the most important technique for neuromodulation. Deep brain stimulation, as the most prescribed neuromodulation method clinically, has been used for treating neurological and psychiatric disorders, such as Parkinson’s Disease, depression, and epilepsy^6–8^. However, the spatial resolution of electrical stimulation is limited by the spread of the electric current, which could distribute over several millimeters and outside of the area of targeting^9^. Providing high spatial precision and cell specificity, optogenetics has been shown as a powerful method of modulating population neural activities in rodents^10,11^. Yet, the requirement of viral infection makes it challenging to be applied in humans. Toward non-genetic stimulation, photothermal neural stimulations based on light absorption of water have been reported^12–14^, and it has attracted increasing interest in basic science and translational application^15,16^. In infrared photothermal neural stimulation (INS), near-infrared light between 1.5 and 2 μm in wavelength is delivered through a fiber and converted into temperature increase in water with sub-millimeter precision^15,17^, where the associated heating raises a significant concern of tissue damage^18^. As a rapidly growing modality, focused ultrasound has been harnessed in a myriad of brain neuromodulation applications^19–21^, given its non-invasive nature with a deep penetration depth^22^. However, ultrasound, with a focus limited by the acoustic wave diffraction, offers a limited spatial resolution at the level of several millimeters^19^, which hinders the study of specific brain regions. In addition, since the ultrasound field easily disrupts the gigaohm seals^23^, it is challenging to integrate ultrasound stimulation with whole-cell patch-clamp electrophysiology, which is the gold standard technique for high-fidelity analysis of the biophysical mechanisms of neural membrane and ion channels^24^.

Our team recently developed a fiber-based optoacoustic converter, which exploited the optoacoustic effect^25^, absorbing pulsed light and producing an ultrasound wave, and achieved neural stimulation in vitro and in vivo at submillimeter spatial resolution^26^. Yet, such resolution is still insufficient for targeting subtypes of neurons at single-cell level or sub-cellular structures. In addition, the device does not allow stable integration with patch clamp on the same cell being stimulated. New capabilities, including single and subcellular precision and integration of single-cell electrophysiology recording, are still sought to enable understanding of mechanical stimulation at the single-cell level and to offer high precision for potential clinical applications.

Here, we report a miniaturized tapered fiber optoacoustic emitter (TFOE) capable of generating an ultrasound field with a 2.7 MPa pressure and a spatial resolution of 39.6 µm, which offers an unprecedented high spatial resolution for ultrasound stimulation. The significant advancement of TFOE in both spatial resolution and optoacoustic conversion efficiency are achieved based on the following innovative designs. First, instead of using a commercial multimode fiber with a diameter of 200 µm as in our earlier work, we developed a controlled tapering strategy and reproducibly tapered the fibers to a tip diameter as small as 20 μm. Second, a new deposition method was developed to achieve uniform and controllable coating thickness of ~10 µm on the small 20-µm fiber tip. Third, instead of using graphite powder in epoxy as a converter, we applied carbon nanotubes (CNT) embedded in a polydimethylsiloxane (PDMS) matrix with improved solubility, which allows highly efficient optoacoustic signal generation from the tapered fiber tip with an increase in the conversion efficiency by one order of magnitude^27^ and prevents light leak from the thin 20 μm coating.

Using TFOE, we improved substantially the spatial and temporal resolution of optoacoustic neuron stimulation. Specifically, we demonstrated single-cell stimulations and subcellular stimulation of axons and dendrites. We also showed that a single acoustic pulse with a sub-microsecond duration was capable to achieve neuron stimulation, which was found as the shortest duration of acoustic stimuli to the best of our knowledge^23^. Importantly, the near field acoustic wave generated by TFOE allowed optoacoustic stimulation with simultaneously monitoring cell responses using whole-cell patch-clamp recording, which had been reported as a challenge for traditional ultrasound^23^. Our studies revealed cell-type-specific responses to acoustic stimulation for excitatory and inhibitory neurons. These advances show the exciting potential of TFOE as a platform technology for non-genetic high-precision stimulation of the neural system, and as a tool for investigations into the mechanisms of ultrasound neural stimulation.

## Results

### Fabrication of TFOE and characterization of acoustic generation

**Fig. 1:**
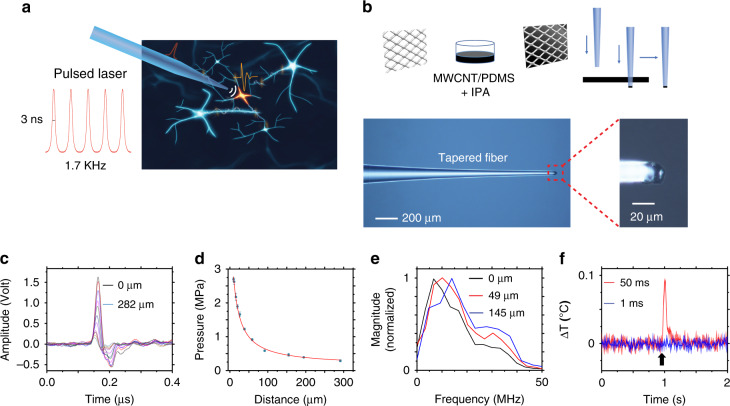
TFOE for high-precision optoacoustic stimulation. TFOE for high-precision optoacoustic stimulation.**a** Schematic of TFOE enabling single-neuron stimulation. **b** Multiwall CNT/PDMS mixture as coating material casted on a metal mesh followed by a punch-through method to coat the tapered fiber. Bottom left: optical image of TFOE, Bottom right: zoom-in showing the tip. **c** Acoustic signal waveforms detected by the hydrophone at the distances ranging from 0 to 282 µm from the TFOE. The curves shown from the top to the bottom were obtained at the distances of 0, 2, 6, 10, 17, 29, 49, 82, 145, 185 and 282 µm, respectively. **d** Detected pressure plotted as a function of the distance. **e** Frequency spectra of acoustic signals acquired at 0, 49 and 145 µm, respectively. All measurements in (**c**–**e**) were done with single laser pulse with a pulse energy of 6.7 µJ. **f** Surface temperature of TFOE tip during laser excitation of 50 ms (7.8 mW, Red) and 1 ms (11.4 mW, Blue), respectively. Shaded area in (**f**): standard deviation taken from three measurements from the same TFOE. Black arrow: laser onset.

Towards single-cell modulation, we have fabricated a TFOE with a 20 μm tip diameter as a miniaturized ultrasound source. We took several innovative steps to overcome the challenges associated with the small 20 μm fiber tip. For control of tapering an optical fiber reproducibly, a multimode fiber was gradually pulled from the full diameter of 225 to 20 μm via a thermal tapering technique (see “Methods”). To convert the light energy into acoustic waves with maximum efficiency^28^, we have optimized the absorption/thermal expansion layer, which composes multi-wall CNTs with strong light absorption embedded in PDMS with a high thermal expansion coefficient^27^. To increase optoacoustic conversion efficiency in the tapered fiber and assure minimum light leakage, the optoacoustic CNT/PDMS coating was prepared with a large CNT concentration of 15%, by introducing isopropyl alcohol (IPA) to form IPA-coated CNTs with hydroxyl groups. To overcome the reduced viscosity of PDMS induced by high CNT concentration and IPA, as well as to achieve a uniform and controlled coating thickness on the 20 μm cross-section of the tapered end, a punch-through method (see “Methods”) was deployed (Fig. 1b). The coating thickness was controlled by changing the matrix viscosity via IPA evaporation. The TFOE was further confirmed by optical imaging to have a CNT/PDMS coating of a thickness of 9.5 μm and an overall diameter of 19.8 μm, meeting the needs of single-cell targeting (Fig. 1b bottom).

Next, a 1030 nm nanosecond pulsed laser was delivered to the TFOE to generate optoacoustic signals. The acoustic signals were measured by a 40 µm needle hydrophone (Fig. S1). As shown in Fig. 1c, the acoustic peak-to-peak intensity attenuates significantly as the distance between the hydrophone and the TFOE increases. The measured pressure *P* is plotted as a function of the distance *d* in Fig. 1d, showing a fitting curve of *P* = 50.88/(*d* + 18.89) +0.14 (*R*^2^ = 0.9999, fitting coefficient of determination) and confirming the inverse proportion relationship of the pressure and distance expected for omnidirectional waves. The spatial resolution of the generated acoustic field, defined by the distance where the pressure decreases to 1/e of initial pressure at 0 µm, was found to be 39.6 µm, 5.5 times smaller compared to the acoustic wavelength of 0.22 mm corresponding to the peak frequency of 6.6 MHz in water. We also confirmed that a laser with the same energy delivered to a bare optical fiber without the photoacoustic coating generated negligible signal detected by the hydrophone at the distance of 10 µm (Fig. S2).

The radio frequency spectrum of the measured acoustic waveforms after Fast Fourier Transform (FFT) exhibit peak acoustic frequencies of 6.6, 10.1, and 13.9 MHz at distances of 0, 49, and 145 µm, respectively (Fig. 1e). This frequency range is similar to previous studies^29,30^, in which a peak frequency of 8 MHz was found for a fiber with ~10 µm coating of CNT/PDMS and 20 MHz for ~1 µm fiber coating. Moreover, the peak frequency of the broad band shifts to a higher value as the distance increases. This could be explained by the different decay dynamics of high and low-frequency acoustic waves. For the acoustic waves with lower frequencies and correspondingly longer wavelengths, the TFOE acts as a point source and the propagation of the low-frequency waves is omnidirectional. Therefore, the acoustic intensity for low frequency is expected to attenuate quickly. In contrast, the high-frequency components with wavelengths comparable to the size of TFOE propagate more like planar waves in water, the attenuation is less for high-frequency components. Therefore, we observed a higher peak frequency at the increased distances.

To characterize the thermal profile generated by TFOE in water during acoustic generation, temperature on the fiber tip was measured by a miniaturized ultrafast thermal sensor (DI-245, DataQ, OH, USA) directly in contact with the TFOE tip surface. Two test conditions were used for successful neuron stimulation: first, a laser pulse train of 50 ms, a laser power at 7.8 mW and a repetition rate of 1.7 kHz; second, a laser pulse train of 1 ms, a laser power at 11.4 mW and a repetition rate of 1.7 kHz. As shown in Fig. 1f, the tip surface temperature increased by only 0.093 ± 0.004 °C under the first condition and the increase was not detectable under the second condition. This temperature increase is far below the threshold of thermal-induced neuron modulation (Δ*T* ≥ 5 °C)^31,32^. Collectively, these results demonstrate that TFOE with a tip diameter of 20 μm fabricated serves as a point ultrasound source, producing ultrasound fields with a spatial resolution of 39.6 µm. This unprecedented spatial resolution will enable high-precision stimulation at single-neuron level while minimizing thermal damage and undesired mechanical disruptions.

### TFOE stimulation of primary neurons with single-cell precision

**Fig. 2:**
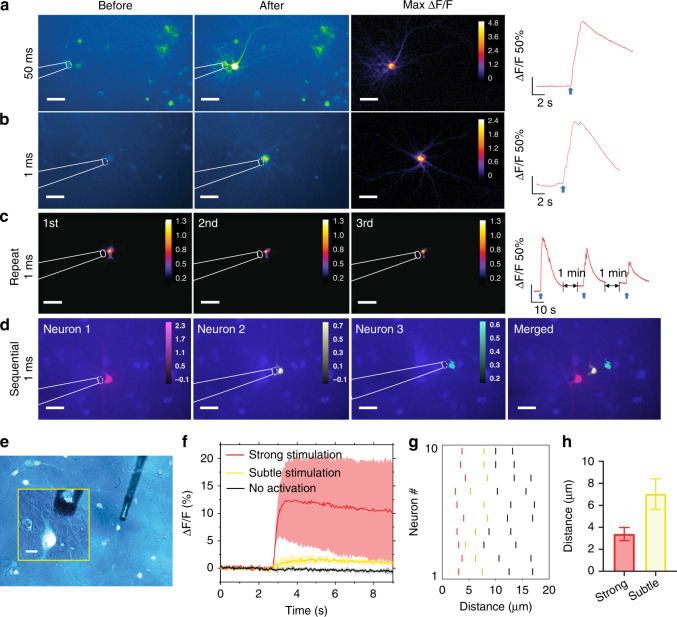
Fluorescence images of GCaMP6f-expressing neurons in response to TFOE stimulation. **a**, **b** Fluorescence images and calcium traces in sparse population stimulated by TFOE with a laser duration of 50 ms (**a**) and 1 ms (**b**). The fluorescence images with the peak intensity after stimulation are shown as “after”. Blue arrows: laser onset. **c** Max ∆*F*/*F* images of one neuron undergone repeated TFOE stimulation three times. Right: calcium signal of the neuron. Blue arrows: laser onset. **d** Sequential stimulation of three neurons. Max ∆*F*/*F* labeled in red, yellow, green respectively superimposed with the fluorescence image. Scale bars: 50 µm. **e** Merged fluorescence image and transmission image of neuron culture. Inset: zoom-in of the targeted neuron and the TFOE tip. Scale bar: 10 µm. **f** Representative fluorescence signals evoked at the targeted neuron by TFOE stimulation. Average Calcium signals induced by strong neuron stimulation when the TFOE was placed at ${\,}<{5}$ µm (Red), subtle stimulation when the TFOE was placed at 5–10 µm (Yellow) and no activation when the distance was ${\,}>{10}$ µm (Black). Shaded areas: the standard deviation from 10 neurons. **g** Calcium response as a function of distance at targeted neurons upon TFOE stimulation. 10 neurons were tested individually. For each neuron, four different distances were tested. Calcium responses were characterized by color bars with the red bars corresponding to the strong stimulation similar to the red curve in (**f**), the yellow bars corresponding to the subtle stimulation and the black bars for no activation. **h** Average distances to evoke strong and subtle activations. Laser condition for (**e**–**h**): 11.4 mW, 1 ms, 1.7 kHz.

To test whether the TFOE provides sufficient spatial precision when modulating a single neuron in culture, we prepared primary rat cortical neurons expressing GCaMP6f and performed calcium imaging using an inverted wide-field fluorescence microscope. Controlled by a micro-manipulator, a TFOE has placed ~5 μm away from a targeted neuron. A 3-ns pulsed laser at 1030 nm and 1.7 kHz repetition rate was used to deliver laser pulses of a 50 millisecond duration at an average power of 7.8 mW, corresponding to 85 pulses. Calcium transients were observed immediately after laser onset for the targeted neurons, while other neurons ~50–70 µm away from the tip remained unaffected (Fig. 2a), indicating high spatial resolution of TFOE stimulation. The calcium transient with max Δ*F*/*F* of 135% ± 83% (*N* = 6 from 3 cultures, data in mean ± SD) indicates successful activation of the targeted neuron likely through the firing of multiple action potentials evoked by TFOE stimulation. To further improve the temporal resolution, a laser pulse train of 1 ms (two pulses) at 11.4 mW power was delivered to the TFOE. Successful activation of single neurons was also observed with a max Δ*F*/*F* of 106% ± 61% (*N* = 8 cells from three cultures, data in mean ± SD) (Fig. 2b).

Figure S3 compared the TFOE stimulation with controls. The control group of 1 ms TFOE with 3 μM tetrodotoxin (TTX) showed no activation, confirming that the calcium increase observed in the experimental groups resulted from Na^+^ channel-dependent action potentials. A laser only group with a pulse train of 1.0 s and 11.4 mW power using a tapered fiber without the coating showed no activation, therefore, the effect of the laser on the neuron activity can be excluded.

We next investigated whether the TFOE can trigger neural activation reliably and repeatedly. Figure 2c shows the fluorescence intensity of the same neuron upon repeated TFOE stimulation for three times. We used 1 ms laser duration for each stimulation and an interval of 1 min between each recording period. Successful activation was achieved for each stimulation on the same neuron, which confirmed the viability of the neuron after TFOE stimulation. A decrease in max Δ*F*/*F* for each sequential stimulation was observed, which could be attributed to calcium depletion^33,34^ or spike frequency adaptation^35^. In addition, we demonstrated the spatial precision of the TFOE stimulation using three neurons selectively targeted by the TFOE. These three neurons had an edge-to-edge spacing of 25 ± 2 μm. The TFOE was sequentially placed about 5 μm away from each of the three targeted neurons. The maximum fluorescence intensity change (Δ*F*/*F*) was color-labeled for each neuron in red, yellow, and green, respectively (Fig. 2d). Importantly, fluorescence increase was observed only for the selectively targeted neuron without simultaneous activation of the other two neurons, indicating that TFOE provided neuron stimulation with single-neuron precision.

To quantify the spatial resolution of the TFOE stimulation, we studied the distance dependence of the TFOE stimulation for the individually targeted neurons. The TFOE was placed at varied distances from the neurons using a micro-manipulator. Transmission imaging was acquired each time prior to fluorescence imaging to visualize the position of TFOE. Figure 2e shows the merged transmission image and fluorescence image of GCaMP-expressing neurons, allowing measurement of the distance between the TFOE and the targeted neuron. For each targeted neuron, the Calcium response was first recorded with the TFOE placed at a distance about 20 µm. Then, the distance was gradually decreased, until strong activations were observed. Fluorescence traces of neuron stimulated by the TFOE at four different distances were recorded. A total of 10 neurons were tested separately. Figure 2f shows three representative Calcium dynamics observed from a targeted neuron, including a typical trace with Δ*F*_max_/*F* > 2%, labeled strong stimulation in red, a subtle signal increase with 0.05% < Δ*F*_max_/*F* < 2% labeled subtle stimulation in yellow, and no activation with Δ*F*_max_/*F* < 0.05% labeled in black. Figure 2g shows the distances where these three types of signal dynamics occurred for all 10 neurons. For the distance <5 µm, the TFOE evokes Calcium transient indicating successful strong activation of the targeted neurons, likely through firing of multiple action potentials. When the TFOE was placed between 5 and 10 µm, subtle Calcium signals were observed, which could result from the Calcium influx through acoustic induced membrane permeabilization^27,36^. At the distance larger than 10 µm, no calcium activation was observed. Considering that Fig. 1d shows that the pressure was 1.9 MPa at the 10 µm distance, this result indicates that TFOE pressure smaller than 1.9 MPa with the 1.7 kHz repetition rate was not sufficient to induce neuron activation. Summarized in Fig. 2h, the average distance to evoke strong and subtle stimulation are 3.4 ± 0.6 µm and 7.0 ± 1.4 µm, respectively. In summary, the TFOE induced neuron activation exhibits a high spatial precision with an effective radius <10 µm.

### Optoacoustic stimulation with a single pulse

**Fig. 3:**
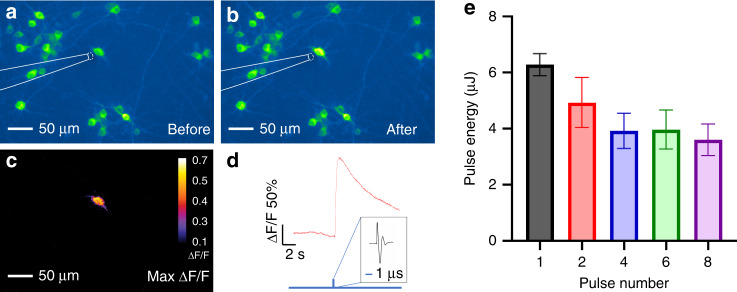
Pulse energy dependence of TFOE stimulation. **a**–**c** Fluorescence images of GCaMP6f expressing neurons before and after TFOE stimulation with a single pulse. **d** Calcium trace of the targeted neuron undergone single-pulse stimulation. Blue vertical line: onset of optoacoustic stimulation with zoom-in showing a representative optoacoustic waveform. **e** Pulse energy threshold for successful neuron stimulation as a function of pulse number (*N* = 5–7).

Taking advantage of the controllability of laser pulse energy and pulse number, we explored the stimulation effect of a single optoacoustic pulse on neurons. The same nanosecond pulsed laser was used to deliver a single laser pulse to the TFOE. TFOE stimulation of the GCaMP6f-expressing primary cortical neuron with different laser pulse energies was performed under the single pulse condition. No calcium transient was observed until the pulse energy reached 6 µJ/pulse (Fig. 3a–d). The width of the optoacoustic wave is <1 μs (Fig. 1c), which is, to the best of our knowledge, the shortest acoustic stimuli for successful neuron modulation so far^23^. This capability could potentially enable acoustic control of neural circuits with unprecedented temporal precision required to mimic natural neural coding^37^.

We further investigated the required laser energy for a given pulse number for successful neuron stimulation. In previous ultrasound studies, continuous wave and pulsed ultrasound with varied intensities and durations have been applied for neuron stimulation^21^. The relationship between temporally-averaged US intensity and response amplitude or success rate was found to be negative^38^ or positive^39^. Given these studies, we pursued a statistical investigation of the behavior of neurons in response to acoustic stimulation across multiple intensities and durations. In our work, first, the threshold of pulse energy for successful stimulation is defined as the laser pulse energy sufficient to induce a maximum fluorescence intensity change (Max Δ*F*/*F*) >20%^40^. The threshold energy shows a monotonic decrease from 6.3 µJ, 4.9 µJ to 3.9 µJ when increasing the pulse number from 1, 2 to 4, respectively, and it remains relatively constant at 3.9 and 3.6 µJ when the pulse number increased to 6 and 8, respectively. These results demonstrated the following key findings. First, the decrease of the energy threshold when the laser pulse number increases in the range of 1–4 shows that under the small pulse energy condition, subthreshold depolarizations accumulate with increasing pulse numbers, consistent with the previous work^41^. Second, the flattening trend of the threshold energy from 4 to 8 pulses implies a presence of an energy threshold at around 4 µJ/pulse, below which the action potential can hardly be evoked with even further elongation of the pulse train. These results are in agreement with previous work^41,42^.

### TFOE stimulation targeting sub-cellular regions of a single neuron

**Fig. 4:**
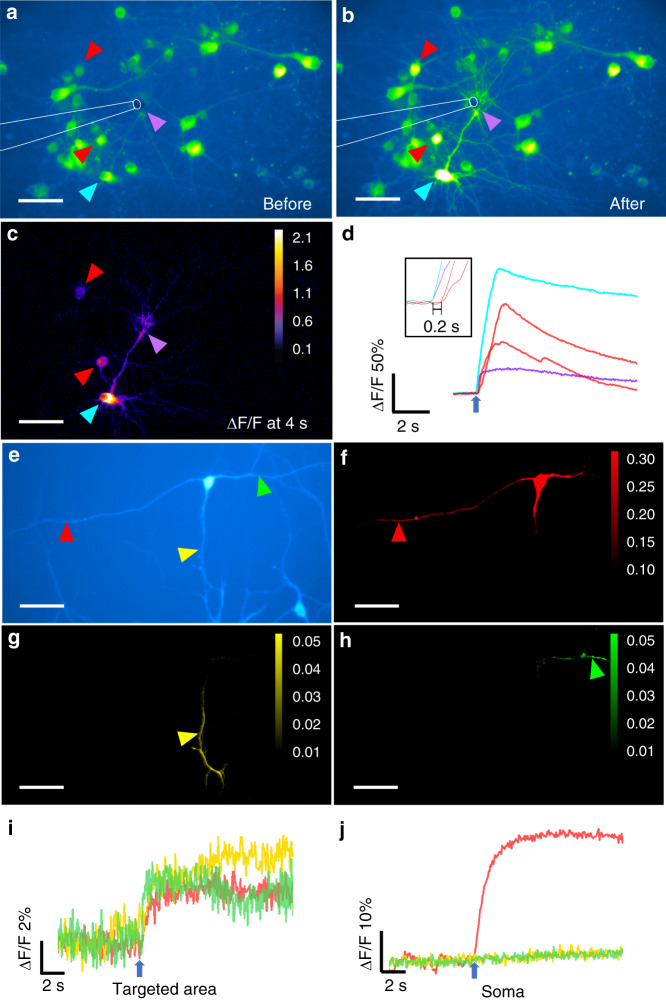
TFOE evoked sub-cellular stimulation on neurites. **a**–**b** TFOE evoked neurites activation with calcium wave propagating along the neuron network. Colored arrows: targeted area (purple), neuron 1 (cyan) and neuron 2, 3 (red). **c** Δ*F*/*F* of calcium signal at 4 s after laser onset. **d** Calcium traces of targeted area (purple), neuron 1 (cyan), and neuron 2 and 3 (red), as labeled in (a). Inset: Zoom-in of calcium signals immediately after the laser onset. Blue arrow: laser onset. **e** A multipolar neuron stimulated with a TFOE selectively targeting the axon (red arrow) and dendrites (yellow and green arrows). **f**–**h** Maximum Δ*F* of calcium signal upon stimulation of different areas. **i** Calcium traces measured at the targeted neurites as labeled in (**e**) by red, yellow and green arrows, respectively. **j** Calcium traces measured at the soma upon stimulation of different neurites. Blue arrows in (**i**–**j**): laser onset. Scale bars in (**a**–**c**), (**e**–**h**): 50 µm

Upon successful stimulation of cultured primary neurons, we further investigated whether the TFOE can target subcellular structures. To this end, the TFOE was first carefully placed above the targeted area where axons and dendrites densely populate without the presence of somas. A 1030-nm laser pulse train with a duration of 1 millisecond, a laser power of 11.4 mW and a repetition rate of 1.7 kHz was delivered to the TFOE. An increase in fluorescence intensity at the targeted area was clearly observed after laser onset, indicating successful TFOE stimulation of targeted neurites (Fig. 4a–b). Three different calcium dynamics were captured through imaging throughout

the field of view. First, a slow propagation of calcium wave initiating from the targeted region in the neural network was observed after TFOE stimulation (Fig. S4). The speed of the calcium wave propagation was calculated to be 75.2 µm/s, which was in agreement with the propagation speed of dendritic calcium waves induced by synaptic activity or by the activity of metabotropic glutamate receptors (mGluRs) and backpropagating action potentials, which generated a speed of ~70 µm/s^43^. Second, four sites in the field of view showed elevated fluorescence signals prior to the spreading of calcium waves (Fig. 4c). The neurites in the targeted region (labeled purple in Fig. 4a–c) and a specific neuron 1 (labeled cyan in Fig. 4a–c) with axon directly connecting to neurites in the targeted region showed fast calcium transients immediately after laser onset (Fig. 4d), which resembles the backpropagation of action potentials. Considering that an unmyelinated axon would conduct action potential spikes at a speed of 500 µm/ms to synapses^44^, the propagation from neurites to neuron 1 (cyan in Fig. 4a–c) over a distance of ~100 µm only requires 0.2 ms. Therefore, the difference in the calcium transient onset for neuron 1 (cyan in Fig. 4d) and the targeted area (purple in Fig. 4d) was non-detectable by the camera with a sampling interval 50 ms. Third, neuron 2 and 3 (labeled red in Fig. 4a–c) in the vicinity but without axons connecting to the targeted area showed an activation delay of ~0.2 s (Fig. 4d, inset) with similar temporal dynamics. This signaling was likely attributed to action potential evoked through synaptic transmission, since it showed a faster propagation speed than the calcium wave.

This capability of TFOE induced stimulation on subcellular structures, specifically on axons and dendrites, was then utilized to elucidate whether axons and dendrite have distinct response profiles to optoacoustic stimulation. In Fig. 4e, three neurites in a multipolar neuron were targeted selectively by the TFOE. Targeted TFOE stimulation on one of the neurites (red in Fig. 4e, f) induced strong calcium activation at the soma with no delay (Fig. 4f, j). Thus, this neurite is identified as an axon, since such propagating activation resembles backpropagation of action potentials in an axon. Distinctively, targeted TFOE stimulation of the other two neurites (yellow and blue in Fig. 4e, g and h) did not induce any activation at the soma (Fig. 4g–j). Thus, they were identified as dendrites. Neuronal dendrites are known to integrate synaptic inputs from upstream neurons, which involves summation of stimuli that arrive in rapid succession, entailing the aggregation of inputs from separate branches. In our case, the forward propagation of a single dendrite was found to be insufficient to evoke action potentials at the soma. The differences between responses of the axon and dendrites upon acoustic stimulation at the single-cell level are shown to be repeatable across multiple neurons (Fig. S5). Collectively, these data reveal differential response dynamics of axons and dendrites to optoacoustic stimulation for the first time, enabled by subcellular targeting capability of TFOE.

### Whole-cell patch-clamp recording reveals cell-type-specific response to TFOE stimulation

A key advantage of single-neuron TFOE stimulation is the compatibility with intracellular patch-clamp recordings. While the calcium response to the stimulation has limited temporal resolution, direct recordings using intracellular patch-clamp recordings stand as the gold standard to study sub- and supra-threshold neuron activity. Conventional ultrasound easily disrupts the patch attachment between the glass and membranes, so intracellular patch-clamp recordings have been challenging during conventional ultrasound stimulation. Our optoacoustic stimulation has the advantage of being highly precise with a minimized mechanical disruption; therefore, it can be recorded with patching, providing a new testing system to gain insights towards mechanical modulation of neural systems.

**Fig. 5:**
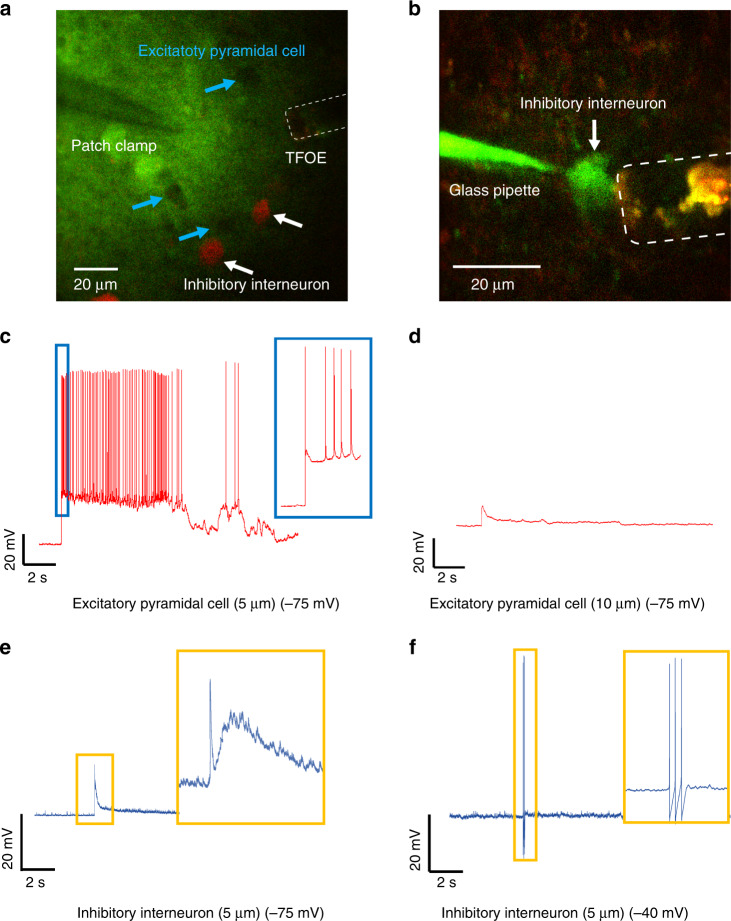
Single neuron patch clamp with TFOE stimulation. **a**–**b** Two-photon imaging of patch clamp integrated with TFOE in a mouse brain slice targeting GAD2-tdTomato negative pyramidal neurons and GAD2-tdTomato positive inhibitory interneurons. The patch pipette is visualized using the cyan-green fluorescent dye Alexa Fluor 488 in the intracellular electrode solution. **c**, **d** Membrane voltage response in an excitatory pyramidal cell upon TFOE stimulation (5 ms) at a distance of ~5 µm (**c**) and ~10 µm (**d**). **e**, **f** Voltage response in an inhibitory interneuron upon TFOE stimulation at ~5 µm at the membrane voltages of −75 mV (**e**) and −40 mV (**f**). Laser: 11.4 mW, 1.7 kHz, 5 ms duration.

We integrated TFOE stimulation with patch-clamp recording on single neurons in mouse cortical slices to detect the direct electrical response to optoacoustic single-neuron stimulation. As shown in Fig. 5a, we used brain slices from mice expressing tdTomato in GAD2 interneurons to assist in visualization of specific cell types. Thus, GAD2-tdTomato positive inhibitory interneurons and GAD2-tdTomato negative pyramidal neurons can be selectively targeted. TFOE can be integrated with the patch pipette to induce depolarization leading to action potential generation in the targeted neurons. Also indicated in Fig. 5c–f, the neuron membrane voltage can be measured precisely with an unprecedented stability upon TFOE stimulation.

For excitatory pyramidal cells, under the current-clamp mode, a train of action potential was observed immediately after TFOE stimulation at 5 µm (Fig. 5c). The result was consistent with previous calcium imaging with Δ*F*/*F* >100% in fluorescence change (Figs. 2a–b and 3d). When the TFOE was moved from 5 to 10 µm away from the neurons (Fig. 5c, d), the action potentials give way to a subthreshold depolarization, indicating a high precision of the stimulation in the tissue.

Next, we targeted tdTomato positive interneurons. TFOE induced subthreshold depolarization in inhibitory interneurons held at −75 mV, and the electrical response over time after stimulation showed two components (Fig. 5e, inset). The first sharp peak could be due to the direct interruption of the membrane integrity by the acoustic wave, and the following broad peak is likely due to an inward channel current, thus indicates the possible involvement of ion channels. With the membrane depolarized via injecting positive currents to near −40 mV, a short train of three action potentials was observed upon TFOE stimulation (Fig. 5f). The distinct response of excitatory pyramidal neuron and inhibitory interneurons to acoustic stimulation is likely contributed by multiple factors including a unique intrinsic action potential threshold of these two cell types, as well as distribution of mechanosensitive ion channels that have different response dynamics to acoustic radiation force^45,46^. In summary, the TFOE provides an unprecedented stable ultrasound source compatible with patch-clamp recordings, holding promise to shed light on the mechanism of acoustic induced neuron stimulation.

## Discussion

In this study, we develop a TFOE that generates acoustic waves with a spatial resolution of 39.6 µm, enabling optoacoustic neural modulation with single-neuron and subcellular precision.

The acoustic wave generated by TFOE allows optoacoustic stimulation along with simultaneous monitoring of cell responses using whole-cell patch-clamp recording, which has been reported to be challenging under conventional ultrasound stimulation. Coupling TFOE with ex vivo brain slice electrophysiology, we revealed cell-type-specific responses to acoustic stimulation for excitatory and inhibitory neurons.

The optoacoustic effect has been extensively used for biomedical imaging^25^, and more recently, it has been explored for neuromodulation^26^. Compared to the previously reported optoacoustic stimulator, the TFOE offers new capabilities through adapting new device designs and innovative fabrication methods. The highly efficient optoacoustic conversion layer in the TFOE is made of CNTs of improved solubility embedded in a thermo-expansive PDMS matrix, which significantly improves light to sound conversion efficiency^27^. In addition, the punch-through coating method ensures uniform coating of the much smaller tapered fiber tip with great control and reliability.

A key advantage of the TFOE stimulation is its unprecedented spatial resolution. Transcranial ultrasound neuromodulation has been demonstrated in rodents^20^, non-human primates^47^, and in humans^19^. However, due to the wave diffraction limit, focused ultrasound neuromodulation offers a spatial precision of a few millimeters^19^, which prohibits site-specific modulation in small animals or single-neuron stimulation and therefore lacks the capabilities to study cell-type-specific responses. The spatial resolution of the TFOE generated acoustic field was found to be 39.6 µm, 5.5 times smaller compared to the acoustic wavelength of 0.22 mm corresponding to the peak frequency of 6.6 MHz in water. Utilizing the generated acoustic field, we demonstrate neural stimulation with single cell and subcellular precision, and reveal the differential response to TFOE stimulation of subcellular structures by specifically targeting the neuronal soma, dendrites, and axons.

By harnessing the controllability of the pulsed laser, we identified the accumulative effect of optoacoustic stimulation at the single-cell level, indicating that ultrasonic stimulus can be integrated over a finite duration to become effective. A previous study by Tyler et al. using ultrasound with focal size of 2 mm, the relationship between the temporal-averaged US intensity and the success rate was found to be negative^38^. This differs from Mourad et al.’s study with ultrasound focal size of 1 mm, where positive relationship was reported^39^. These studies provide conflicting evidence^21^, where the observed behaviors may due to changes in ultrasound parameters, or selective modulation of specific regions. In another study by Pauly et al., an ultrasound with a focal spot of 4 mm in diameter was used to induce short-latency muscle contractions in mice measured by electromyography (EMG), showing the ultrasonic stimulus could be integrated over time, and the presence of energy threshold was demonstrated, below which the response could not be evoked with even further elongation of the stimulation duration^41^. The result by Pauly et al. is consistent with the TFOE data. In addition to intrinsic cell properties, their observations could originate from non-specific neural network targeting and recording. The result from TFOE with the capability of assessing single-neuron activity in a network-free condition further ascertains the stimulus accumulation effect as an intrinsic signal interpretation of individual neurons.

More importantly, successful TFOE stimulation has been achieved with a single laser pulse of 3 ns, which generates an acoustic pulse of sub-microsecond. Previously, single tone burst ultrasound with 10 acoustic cycles and overall duration of 22.7 μs has been reported as the shortest acoustic stimuli for neuron modulation^23^. Therefore, our result represents significant improvement of temporal resolution of current acoustic stimulation techniques.

Furthermore, TFOE allows integration of acoustic stimulation with whole-cell patch-clamp recordings. Our electrophysiological recordings of TFOE stimulated single neurons in brain slices revealed distinct responses of excitatory pyramidal neurons and inhibitory interneurons to TFOE stimulation. The distinct responses may originate from differences in the intrinsic threshold or variations in the distribution of different ion channels. Moreover, the inhibitory neurons showed an elevated threshold of action potential generation compared to excitatory neurons. This contrasts with findings using electric stimulation, where the inhibitory neurons could have a lower threshold than pyramidal cells^48,49^. This discrepancy can be attributed to the different mechanisms between acoustic and electric stimulation. Several hypotheses have been proposed for ultrasound neurostimulation, including the activation of mechanosensitive ion channels^50–53^, the transient mechanical disruption of the neural membrane including the opening of pores^54^, and induction of capacitive currents by intramembrane cavitation^54,55^. In our study, the TFOE evokes electrical response results (Fig. 5e), indicating that two mechanisms, specifically, transient disruption of the membrane and activation of ion channels, are possibly involved. Future systematic studies, for example, patch-clamp recording of TFOE stimulated neurons with genetically or pharmaceutically modification of the ion channels could offer deeper insights of the more detailed mechanism^56^.

In summary, this genetic-free, single-cell stimulation technique offers a new tool to understand the mechanism of neuron stimulation. Moreover, for clinical application, TFOE can be used as a surgical tool or an implant for precise stimulation of a single nerve. For example, TFOE can be used to assist the SDR surgery. In a SDR surgery, precise stimulation of the individual dorsal root nerves is needed to identify the abnormal one. Due to current spread, the commonly used electric stimulation lacks sufficient spatial precision desired as the dorsal root nerves can be as small as 0.27 ± 0.13 mm^57^. Such precision is even more challenging in children patients with cerebral palsy, as their nerves are finer. TFOE provides superior stimulation precision needed. For fundamental studies, the TFOE approach offers a non-genetic neural stimulation method with a tunable spatial resolution through varying the fiber diameters. For example, mapping the organization and connectivity of columns in primates requires precise stimulation of single columns, roughly 200 μm in sizes. Compared to the electrical stimulation, TFOE offers a spatial precision finer than 200 μm and MRI compatibility, making it possible to assess functional outcome of the stimulation through MRI-guided insertion^58^ and brain fMRI recording in humans and non-human primates. It is also non-genetic, which overcoming the challenges facing by optogenetics in primates. This capacity of TFOE will enable mapping of columnar connectome in cortex, which will build a foundation for further development of an optoacoustic brain-machine interface.

## Materials and methods

### Optical fiber tapering

To control the tapering, a multimode fiber (FT200EMT, Thorlabs, Inc., NJ, USA) was pulled at one end by a traction weight with the other end fixed. The pulling force, determined by the weight of the traction object, was found to be proportional to the square of the tapered end radius, therefore used as the key parameter to control the diameter of the tapered end. In this way, with a pulling force of 0.75 N, tapered fibers of 18.4 ± 0.9 μm (*N* = 5) in diameter were fabricated with high reproducibility.

### Tapered fiber coating

To assure a maximum optoacoustic conversion efficiency and minimum light leakage in the tapered fiber, CNT/PDMS/IPA composite was prepared. For PDMS, the silicone elastomer (*Sylgard* 184, Dow Corning Corporation, USA) was dispensed directly into a container carefully to minimize air entrapment, followed by mixing with the curing agent in a ratio of 10:1 by weight. Multiwall CNTs (<8 nm OD, 2–5 nm ID, length 0.5–2 µm, VWR, Inc., NY, USA) and IPA were added to PDMS. The mixture was sonicated for 5 min followed by degassing in vacuum for 30 min. Considering the evaporation of IPA, the final CNT concentration in PDMS reached to 15%. The coating matrix was then casted on a metal mesh to form a uniform film. After partial evaporation of the IPA at room temperature for 10 min, the fiber was controlled by a 3-D micromanipulator to punch through the film with a layer transferred to the tapered end. The coated fiber was then cured vertically at 100 °C.

### Optoacoustic signal measurement

A customized and compact passively Q-switched diode-pumped solid-state laser (1030 nm, 3 ns, 100 μJ, repetition rate of 1.7 kHz, RPMC, Fallon, MO, USA) was used as the excitation source. The laser was connected to an optical fiber through a homemade fiber jumper (SMA-to-SC/PC, ~81% coupling efficiency), then connected to the TFOE with a SubMiniature version A (SMA) connector. To adjust the laser power, fiber optic attenuator sets (multimode, varied gap of 2/4/8/14/26/58 mm, SMA Connector, Thorlabs, Inc., NJ, USA) were used. A needle hydrophone (ID. 40 µm; OD, 300 µm) with a frequency range of 1–30 MHz (NH0040, Precision Acoustics Inc., Dorchester, UK) was utilized for the acoustic measurement. The acquired signal was processed with an ultrasonic pre-amplifier (0.2–40 MHz, 40 dB gain, Model 5678, Olympus, USA) and a digital oscilloscope (DSO6014A, Agilent Technologies, CA). The distance between the TFOE tip and hydrophone was controlled from 0 to 282 µm using a 4-axis micro-manipulator (MC1000e controller with MX7600R motorized manipulator, Siskiyou Corporation, OR, USA) with a controllable motion of 0.2 µm. The distance was measured using a widefield microscope with a 20× objective. The TFOE tip and hydrophone tip were both immersed in degassed water dropped on a cover glass. The setup of the measurement is shown in Fig. S1. The pressure values were calculated based on the calibration curve obtained from the hydrophone manufacturer. The frequency data was obtained through the FFT using Origin 2019.

### Embryonic neuron culture

All experimental procedures have complied with all relevant guidelines and ethical regulations for animal testing and research established and approved by the Institutional animal care and use committee of Boston University (PROTO201800534). Primary cortical neuron cultures were derived from Sprague-Dawley rats. Cortices were dissected out from embryonic day 18 (E18) rats of either sex and digested in papain (0.5 mg/mL in Earle’s balanced salt solution) (Thermo Fisher Scientific Inc., MA). Dissociated cells were washed with and triturated in 10% heat-inactivated fetal bovine serum (FBS, Atlanta Biologicals, GA, USA), 5% heat-inactivated horse serum (HS, Atlanta Biologicals, GA, USA), 2 mM Glutamine-Dulbecco’s Modified Eagle Medium (DMEM, Thermo Fisher Scientific Inc., MA, USA), and cultured in cell culture dishes (100 mm diameter) for 30 min at 37 °C to eliminate glial cells and fibroblasts. The supernatant containing neurons was collected and seeded on poly-D-lysine coated cover glass and incubated in a humidified atmosphere containing 5% CO_2_ at 37 °C with 10% FBS + 5% HS + 2 mM glutamine DMEM. After 16 h, the medium was replaced with Neurobasal medium (Thermo Fisher Scientific Inc., MA, USA) containing 2% B27 (Thermo Fisher Scientific Inc., MA, USA), 1% N2 (Thermo Fisher Scientific Inc., MA, USA), and 2 mM glutamine (Thermo Fisher Scientific Inc., MA, USA). Cultures were treated with 5 µM FDU (5-fluoro-2′-deoxyuridine, Sigma-Aldrich, MO, USA) at day 5 in culture to further reduce the number of glial cells. AAV9.Syn.Flex.GCaMP6f.WPRE.SV40 virus (Addgene, MA, USA) was added to the cultures at a final concentration of 1 μl/ml at day 5 in culture for GCaMP6f expressing. Half of the medium was replaced with fresh culture medium every 3–4 days. Cells cultured in vitro for 10–13 days were used for TFOE stimulation experiment.

### In vitro neurostimulation

A Q-switched 1030-nm nanosecond laser (Bright Solution, Inc. Calgary Alberta, CA) was used to deliver light to the TFOE. A 3-D micromanipulator (Thorlabs, Inc., NJ, USA) was used to position the TFOE approaching the cells. Calcium fluorescence imaging was performed on a lab-built wide-field fluorescence microscope based on an Olympus IX71 microscope frame with a 20× air objective (UPLSAPO20X, 0.75NA, Olympus, MA, USA), illuminated by a 470 nm LED (M470L2, Thorlabs, Inc., NJ, USA) and a dichroic mirror (DMLP505R, Thorlabs, Inc., NJ, USA). Image sequences were acquired with a scientific CMOS camera (Zyla 5.5, Andor) at 20 frames per second. Neurons expressing GCaMP6f at DIV (day in vitro) 10–13 were used for stimulation experiment. For TTX control group, tetrodotoxin citrate (ab120055, Abcam, MA, USA) was added to the culture to reach 3 μM final concentration 10 min before Calcium imaging. The fluorescence intensities, data analysis, and exponential curve fitting were analyzed using ImageJ (Fiji), Origin 2019, and GraphPad Prism 8.

### Ex vivo whole-cell patch clamp

All experimental protocols were approved by the Boston University Institutional Animal Care and Use Committee (PROTO201800599). GAD2-Cre/tdTomato mice (The Jackson Laboratory, ME, USA) were used for visualization of inhibitory interneurons. Brain slices were prepared from mice aged post-natal day 60 or greater (both genders). After anesthetization with isoflurane and decapitation, brains were removed and immersed in 0 °C solution of standard artificial cerebral spinal fluid (ACSF). For recordings, slices were moved to the stage of a two-photon imaging system. All recordings were conducted between 33 and 36 °C. Standard patch-clamp solutions and electrodes with resistances between 3 and 4 MΩ were used. The electrode pipette was visualized using the cyan-green fluorescent dye Alexa Fluor 488 hydrazide (Thermo Fisher Scientific Inc., MA, USA), which was added to the intracellular electrode solution (0.3% weight/volume). Imaging was performed using a two-photon imaging system (Thorlabs, Inc., NJ, USA) with a mode-locked Ti:Sapphire laser (Chameleon Ultra II; Coherent, CA, USA) set to wavelengths between 920 nm and 950 nm, which was used to excite both the Alexa Fluor 488 and tdTomato using a 20×, NA 1.0 objective lens (Olympus, MA, USA). Laser scanning was performed using resonant scanners and fluorescence was detected using two photomultiplier tubes (Hamamatsu, JP) equipped with red and green filters to separate emission from Alexa Fluor 488 and tdTomato. All other procedures were following our past work^59^.

### Data analysis

Calcium images were analyzed using ImageJ. Calcium traces, electrophysiological traces were analyzed and plotted using Origin and GraphPad Prism. All statistical analysis was done using Origin. Data shown are mean ± SD.

## Supplementary information

Supplementary information

## References

[1] Houweling AR, Brecht M (2008). Behavioural report of single neuron stimulation in somatosensory cortex. Nature.

[2] Li C-YT, Poo M-m, Dan Y (2009). Burst spiking of a single cortical neuron modifies global brain state. Science.

[3] Rizzo JF (2011). Update on retinal prosthetic research: the Boston Retinal Implant Project. J. Neuro-Ophthalmol..

[4] Palanker D (2005). Design of a high-resolution optoelectronic retinal prosthesis. J. Neural Eng..

[5] Grunt S, Becher JG, Vermeulen RJ (2011). Long‐term outcome and adverse effects of selective dorsal rhizotomy in children with cerebral palsy: a systematic review. Dev. Med. Child Neurol..

[6] Boon P (2007). Deep brain stimulation in patients with refractory temporal lobe epilepsy. Epilepsia.

[7] Mayberg HS (2005). Deep brain stimulation for treatment-resistant depression. Neuron.

[8] Rosin B (2011). Closed-loop deep brain stimulation is superior in ameliorating parkinsonism. Neuron.

[9] Ineichen C, Shepherd NR, Sürücü O (2018). Understanding the effects and adverse reactions of deep brain stimulation: is it time for a paradigm shift toward a focus on heterogenous biophysical tissue properties instead of electrode design only?. Front. Hum. Neurosci..

[10] Boyden ES (2005). Millisecond-timescale, genetically targeted optical control of neural activity. Nat. Neurosci..

[11] Kim CK, Adhikari A, Deisseroth K (2017). Integration of optogenetics with complementary methodologies in systems neuroscience. Nat. Rev. Neurosci..

[12] Wells J (2005). Optical stimulation of neural tissue in vivo. Opt. Lett..

[13] Wells J (2007). Biophysical mechanisms of transient optical stimulation of peripheral nerve. Biophys. J..

[14] Izzo AD (2006). Laser stimulation of the auditory nerve. Lasers Surg. Med..

[15] Cayce JM (2014). Infrared neural stimulation of primary visual cortex in non-human primates. Neuroimage.

[16] Cayce JM (2015). Infrared neural stimulation of human spinal nerve roots in vivo. Neurophotonics.

[17] Xu AG (2019). Focal infrared neural stimulation with high-field functional MRI: a rapid way to map mesoscale brain connectomes. Sci. Adv..

[18] Chernov MM, Chen G, Roe AW (2014). Histological assessment of thermal damage in the brain following infrared neural stimulation. Brain Stimulation.

[19] Legon W (2014). Transcranial focused ultrasound modulates the activity of primary somatosensory cortex in humans. Nat. Neurosci..

[20] Tufail Y (2011). Ultrasonic neuromodulation by brain stimulation with transcranial ultrasound. Nat. Protoc..

[21] Blackmore J (2019). Ultrasound neuromodulation: a review of results, mechanisms and safety. Ultrasound Med. Biol..

[22] Naor O, Krupa S, Shoham S (2016). Ultrasonic neuromodulation. J. Neural Eng..

[23] Tyler WJ (2008). Remote excitation of neuronal circuits using low-intensity, low-frequency ultrasound. PLoS ONE.

[24] Kodandaramaiah SB (2012). Automated whole-cell patch-clamp electrophysiology of neurons in vivo. Nat. Methods.

[25] Wang LV, Hu S (2012). Photoacoustic tomography: in vivo imaging from organelles to organs. Science.

[26] Jiang Y (2020). Optoacoustic brain stimulation at submillimeter spatial precision. Nat. Commun..

[27] Shi L (2020). A fiber optoacoustic emitter with controlled ultrasound frequency for cell membrane sonoporation at submillimeter spatial resolution. Photoacoustics.

[28] Noimark S (2018). Polydimethylsiloxane composites for optical ultrasound generation and multimodality imaging. Adv. Funct. Mater..

[29] Noimark S (2016). Carbon‐nanotube–PDMS composite coatings on optical fibers for all‐optical ultrasound imaging. Adv. Funct. Mater..

[30] Poduval RK (2017). Optical fiber ultrasound transmitter with electrospun carbon nanotube-polymer composite. Appl. Phys. Lett..

[31] Lyu Y (2016). Semiconducting polymer nanobioconjugates for targeted photothermal activation of neurons. J. Am. Chem. Soc..

[32] Shapiro MG (2012). Infrared light excites cells by changing their electrical capacitance. Nat. Commun..

[33] Cohen JE, Fields RD (2004). Extracellular calcium depletion in synaptic transmission. Neuroscientist.

[34] Asteriti S, Liu C-H, Hardie RC (2017). Calcium signalling in *Drosophila* photoreceptors measured with GCaMP6f. Cell Calcium.

[35] Ha GE, Cheong E (2017). Spike frequency adaptation in neurons of the central nervous system. Exp. Neurobiol..

[36] Lin C-R (2010). Sonoporation-mediated gene transfer into adult rat dorsal root ganglion cells. J. Biomed. Sci..

[37] Shemesh OA (2017). Temporally precise single-cell-resolution optogenetics. Nat. Neurosci..

[38] Tufail Y (2010). Transcranial pulsed ultrasound stimulates intact brain circuits. Neuron.

[39] Mehić E (2014). Increased anatomical specificity of neuromodulation via modulated focused ultrasound. PLoS ONE.

[40] Dana H (2018). High-performance GFP-based calcium indicators for imaging activity in neuronal populations and microcompartments. Nat. Methods.

[41] King RL (2013). Effective parameters for ultrasound-induced in vivo neurostimulation. Ultrasound Med. Biol..

[42] Mihran RT, Barnes FS, Wachtel H (1990). Temporally-specific modification of myelinated axon excitability in vitro following a single ultrasound pulse. Ultrasound Med. Biol..

[43] Nakamura T (1999). Synergistic release of Ca2+ from IP3-sensitive stores evoked by synaptic activation of mGluRs paired with backpropagating action potentials. Neuron.

[44] Micheva KD (2016). A large fraction of neocortical myelin ensheathes axons of local inhibitory neurons. Elife.

[45] Kubanek J (2018). Neuromodulation with transcranial focused ultrasound. Neurosurgical Focus.

[46] Tyler WJ (2011). Noninvasive neuromodulation with ultrasound? A continuum mechanics hypothesis. Neuroscientist.

[47] Deffieux T (2013). Low-intensity focused ultrasound modulates monkey visuomotor behavior. Curr. Biol..

[48] Mahmud M, Vassanelli S (2016). Differential modulation of excitatory and inhibitory neurons during periodic stimulation. Front. Neurosci..

[49] Prestigio, C. et al. Spike-related electrophysiological identification of cultured hippocampal excitatory and inhibitory neurons. *Molecular Neurobiology* **56**, 6276–6292 (2019). 10.1007/s12035-019-1506-5 30746640

[50] Kubanek J (2016). Ultrasound modulates ion channel currents. Sci. Rep..

[51] Kubanek J, Shukla P, Das A, Baccus SA, Goodman MB (2018). Ultrasound elicits behavioral responses through mechanical effects on neurons and ion channels in a simple nervous system. J. Neurosci..

[52] Yoo, S., Mittelstein, D. R., Hurt, R. C., Lacroix, J. J. & Shapiro, M. G. Focused ultrasound excites neurons via mechanosensitive calcium accumulation and ion channel amplification. Preprint at https://www.biorxiv.org/content/10.1101/2020.05.19.101196v1 (2020). 10.1038/s41467-022-28040-1 PMC8789820 35078979

[53] Ye J (2018). Ultrasonic control of neural activity through activation of the mechanosensitive channel MscL. Nano Lett..

[54] Krasovitski B, Frenkel V, Shoham S, Kimmel E (2011). Intramembrane cavitation as a unifying mechanism for ultrasound-induced bioeffects. Proc. Natl Acad. Sci. USA.

[55] Plaksin M, Shoham S, Kimmel E (2014). Intramembrane cavitation as a predictive bio-piezoelectric mechanism for ultrasonic brain stimulation. Phys. Rev. X.

[56] Gaub BM (2020). Neurons differentiate magnitude and location of mechanical stimuli. Proc. Natl Acad. Sci. USA.

[57] Liu Y, Zhou X, Ma J, Ge Y, Cao X (2015). The diameters and number of nerve fibers in spinal nerve roots. J. Spinal Cord. Med..

[58] Chen Y (2019). MRI-guided robotic arm drives optogenetic fMRI with concurrent Ca 2+ recording. Nat. Commun..

[59] Fernandez FR, Rahsepar B, White JA (2018). Differences in the electrophysiological properties of mouse somatosensory layer 2/3 neurons in vivo and slice stem from intrinsic sources rather than a network-generated high conductance state. Eneuro.

