# Supplementary material for: Non-genetic photoacoustic stimulation of single neurons by a tapered fiber optoacoustic emitter
Source: Light Sci Appl. 2021 Jul 14;10:143. doi: 10.1038/s41377-021-00580-z (PMC8277806; doi:10.1038/s41377-021-00580-z)
Supplement: Supplementary file 1 — Supplementary information [file 41377_2021_580_MOESM1_ESM.docx]

Supplementary Information for

Non-genetic photoacoustic stimulation of single neurons by a tapered fiber optoacoustic emitter

Linli Shi^1#^, Ying Jiang^2#^, Fernando R. Fernandez^2,5,6^, Lu Lan^3^, Guo Chen^3^, Heng-ye Man^4,5^, John A. White^2,5,6^, Ji-Xin Cheng^2,3*^, Chen Yang^1, 3*^

*^1^ Department of Chemistry, Boston University, 580 Commonwealth Avenue, Boston, MA 02215, USA*

*^2^ Department of Biomedical Engineering, Boston University, 44 Cummington Mall, Boston, MA 02215, USA*

*^3^ Department of Electrical and Computer Engineering, 8 St. Mary’s Street, Boston, MA 02215, USA*

*^4^ Department of Biology, Boston University, 5 Cummington Mall, Boston, MA, 02215, USA*

*^5^ Center for Systems Neuroscience, Boston University, 610 Commonwealth Ave, Boston, MA 02215, USA*

*^6^ Neurophotonics Center, Photonics Center, Boston University, 8 St. Mary’s Street, Boston MA 02215, USA*

***#*** *equal contributions.*

******* *Corresponding author: [jxcheng@bu.edu](mailto:jxcheng@bu.edu),* [*cheyang@bu.edu*](mailto:cheyang@bu.edu)


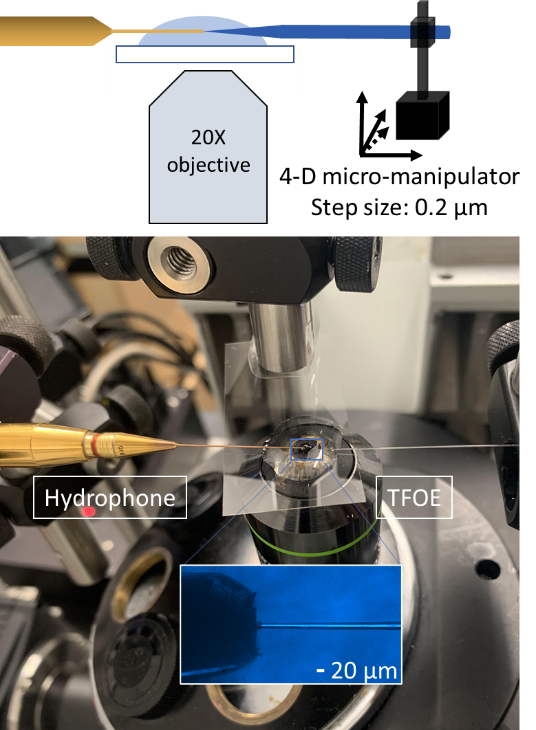


**Fig. S1. Experimental set up for optoacoustic characterization.** Inset: optical image of the hydrophone and TFOE.


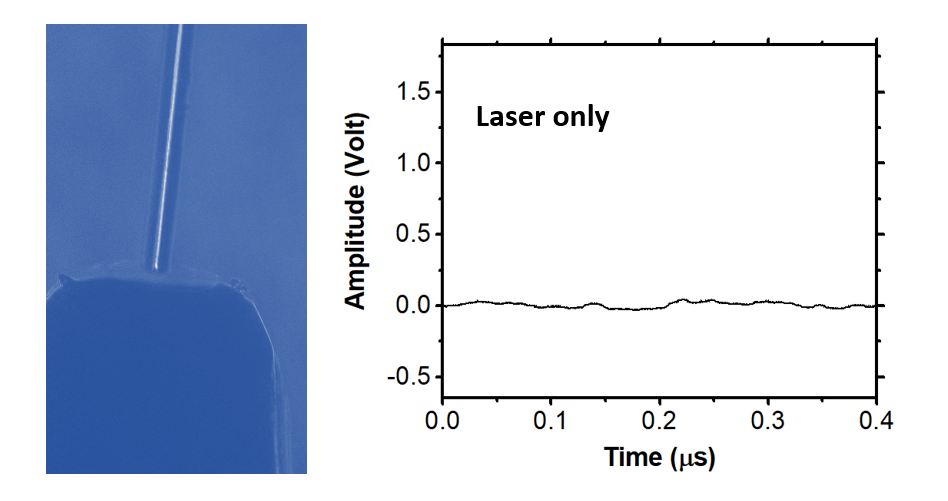


**Fig. S2. Influence of laser on the hydrophone using a bare tapered fiber.** Laser pulse energy: 6.7 µJ.


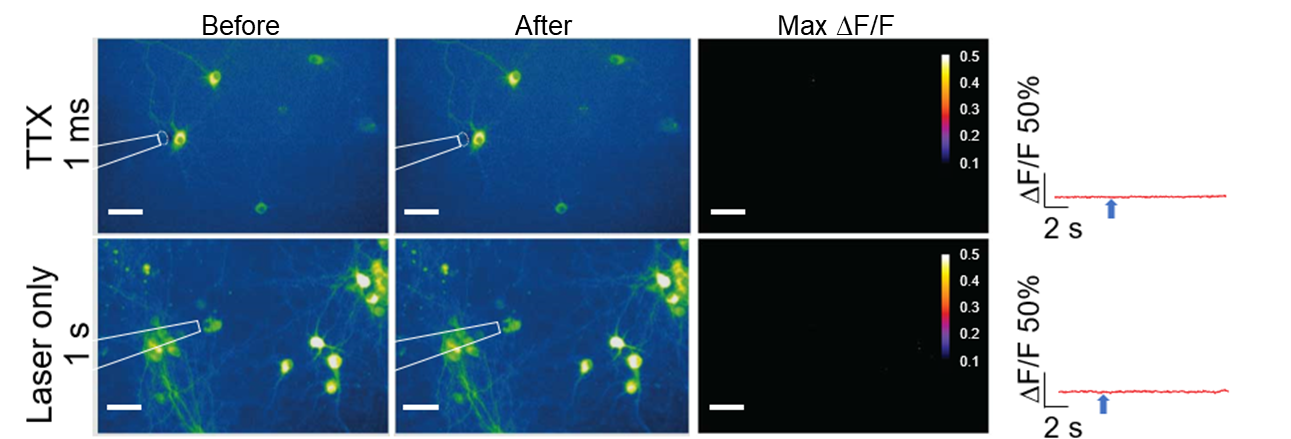


**Fig. S3. Fluorescence images and calcium traces of single neurons in sparse population stimulated by TFOE (1 millisecond) with TTX and laser only (1 s).** Laser repetition rate: 1.7 kHz; average power: 11.4 mW. Scale bars: 50 µm. Blue arrows: laser onset.


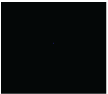

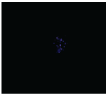

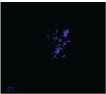

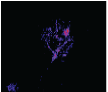

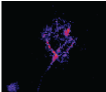

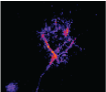


0

.

0

0

s

0

.

1

5

s

0

.

3

0

s

0

.

45

s

0

.

6

0

s

0

.

7

5

s


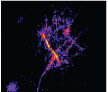


1

.

00

s


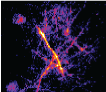

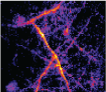


2

.

00

s

9

.

00

s


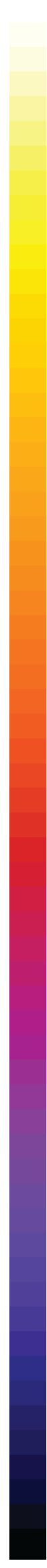


0

1

**Fig. S4. TFOE evoked axon stimulation with calcium wave propagating along neuron network.** Fluorescence contrast images (ΔF/F) were taken at varied time points. White circles: the position of TFOE tip. Laser duration: 1 ms; repetition rate: 1.7 kHz; average power: 11.4 mW. Scale bars: 50 µm.


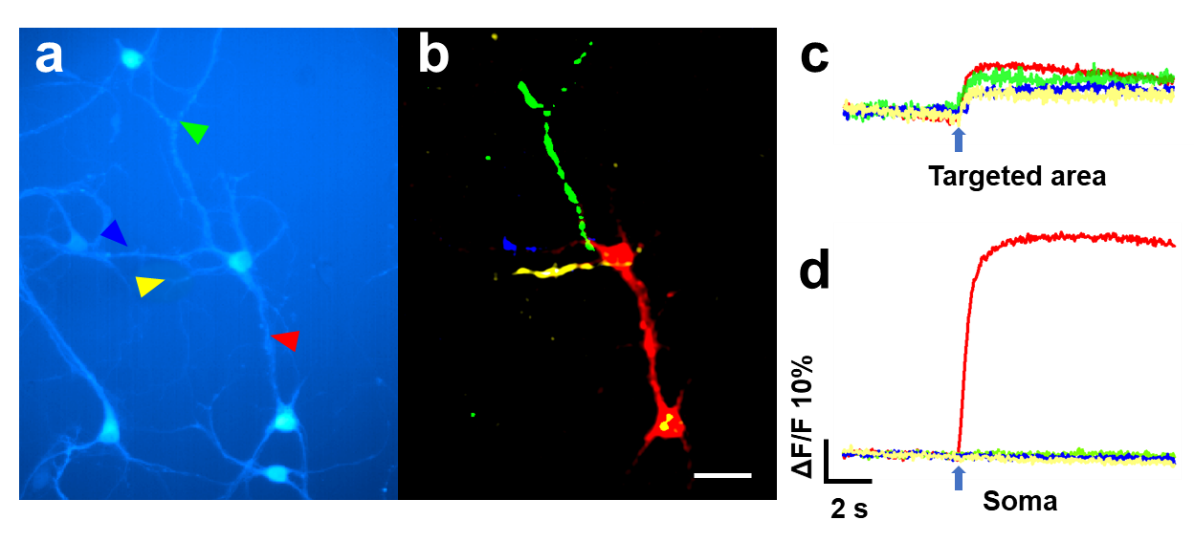
 **Fig. S5. Subcellular spatial precision of TFOE targeting axon and dendrites. (a)** Another multipolar neuron with TFOE targeting axon (red) and dendrites (green, blue and yellow). (**b)** Maximum ΔF of calcium signal upon stimulation of different areas. **(c)** Calcium traces of targeted areas upon stimulation. **(d)** Calcium traces of the soma of targeted neuron upon stimulation of different neurites. Scale bar: 50 µm.
